# Liver-Specific Contrast-Enhanced Magnetic Resonance Cholangio-Pancreatography (Ce-MRCP) in Non-Invasive Diagnosis of Iatrogenic Biliary Leakage

**DOI:** 10.3390/diagnostics13101681

**Published:** 2023-05-09

**Authors:** Renato Argirò, Bruno Sensi, Leandro Siragusa, Luigi Bellini, Luigi Edoardo Conte, Camilla Riccetti, Giovanna Del Vecchio Blanco, Edoardo Troncone, Roberto Floris, Mike Salavracos, Giuseppe Tisone, Alessandro Anselmo

**Affiliations:** 1 Interventional Radiology Unit, Department of Biomedicine and Prevention, Tor Vergata University of Rome, 00133 Rome, Italy; 2 HPB and Transplant Unit, Department of Surgery, Tor Vergata University of Rome, 00133 Rome, Italy; 3 Gastroenterology Unit, Department of System medicine, Tor Vergata University of Rome, 00133 Rome, Italy; 4 Diagnostic Imaging, Department of Biomedicine and Prevention, Tor Vergata University of Rome, 00133 Rome, Italy; 5 Department of Surgery, Cliniques Universitaires St-Luc, 1200 Brussels, Belgium

**Keywords:** bile leak, contrast-enhanced magnetic resonance cholangio-pancreatography, percutaneous transhepatic cholangiography, hepato-biliary surgery

## Abstract

Current non-invasive diagnostic modalities of iatrogenic bile leak (BL) are not particularly sensitive and often fail to localise the BL origin. Percutaneous transhepatic cholangiography (PTC) and endoscopic retrograde cholangiopancreatography (ERCP) are considered the gold standard, yet are invasive studies with potential complications. Ce-MRCP has been not comprehensively studied in this setting but may prove particularly helpful given its non-invasive nature and the anatomical dynamic detail. This paper reports a monocentric retrospective study of BL patients referred between January 2018 and November 2022 submitted to Ce-MRCP followed by PTC. The primary outcome was the accuracy of Ce-MRCP in detecting and localising BL compared to PTC and ERCP. Blood tests, coexisting cholangitis features and time for leak resolution were also investigated. Thirty-nine patients were included. Liver-specific contrast-enhanced MRCP detected BL in 69% of cases. The BL localisation was 100% accurate. Total bilirubin above 4 mg/dL was significantly associated with false negative results of Ce-MRCP. Ce-MRCP is highly accurate in detecting and localising BL, but sensitivity is significantly reduced by a high bilirubin level. Ce-MRCP may be very useful in early BL diagnosis and in accurate pre-treatment planning, but can only be reliably used in selected patients with TB < 4 mg/dL. Non-surgical techniques, both radiological and endoscopic, are proven to be effective in terms of leak resolution.

## 1. Introduction

Iatrogenic bile leak (BL) is a rather common complication that can occur after biliary, hepatic and pancreatic surgery. Biliary complications are common after liver transplantation (10–30%), major hepatic surgery, pancreaticoduodenectomy (PD) and cholecystectomy, particularly if the latter is performed laparoscopically (up to 1.2%) [1]. Patient age, white blood cell counts, intraoperative bleeding, duration and type of surgical procedure are all associated with an increased risk of major biliary complications such as stenosis and BL [2].

BL can cause significant morbidity and mortality so it requires prompt diagnosis and treatment. Accurate diagnosis of BL is crucial to optimise post-operative management in an effort to significantly reduce morbidity and mortality related to this condition. However, documenting the presence and extent of BL can be difficult, often requiring multiple investigations before reaching a definitive diagnosis.

Current techniques to evaluate the biliary tree for suspected bile duct injuries include ultrasound (US), computed tomography (CT), magnetic resonance cholangiography-pancreatography (MRCP), chole-scintigraphy, percutaneous transhepatic cholangiography (PTC) and endoscopic retrograde cholangiopancreatography (ERCP).

US and CT can determine the presence of fluid collection, while conventional MRCP is highly sensitive for the diagnosis of anatomic abnormalities but does not provide functional assessment of the biliary tree. Historically, chole-scintigraphy was employed to confirm leaks. Scintigraphy is highly sensitive for leak detection but lacks the anatomical detail necessary for treatment planning. ERCP and PTC are usually necessary to document the exact location of the leak, providing an opportunity for treatment delivery at the same time. Although both techniques are considered minimally invasive, they can be complicated by potentially serious procedure-related adverse events [3,4].

Gadoxetic acid disodium (Gd-EOB-DTPA, Primovist^®^; Bayer) is an intravenous MRI contrast agent that demonstrates hepatocyte uptake and dynamic biliary excretion. Bile enhancement is seen 20 min after intravenous injection of the contrast medium. In the excretory phase of liver-specific contrast-enhanced magnetic resonance cholangiopancreatography (Ce-MRCP), the biliary tree is well depicted, with detailed images that can even demonstrate leaks from small peripheral branches [5].

However, only a few studies have evaluated postoperative biliary leakage using MRCP with Gd-EOB-DTPA [2,6,7,8,9,10,11].

In this context, the purpose of the present study is to assess the value of liver-specific (Gd-EOB-DTPA) Ce-MRCP for the early detection of postoperative BL in a group of patients later studied with PTC and ERCP for further characterisation and treatment of the leakage.

## 2. Materials and Methods

This was a monocentric retrospective study including patients who developed bile leaks as a postoperative complication of hepato-pancreatico-biliary (HPB) surgery at a single tertiary referral centre, Policlinico Tor Vergata.

Patients were identified retrospectively through the hospital’s database. All consecutive patients operated on between 1 January 2018 and 30 November 2022 were eligible for inclusion.

### 2.1. Patient Selection

All patients with an abdominal (either surgical or percutaneous) drainage positive for the presence of bilirubin undergoing Ce-MRCP investigation followed by PTC/ERCP were included.

Exclusion criteria were:

- Patients under 18 years old
- Patients who were pregnant
- Patients who had not undergone both Ce-MRCP and PTC/ERCP.

### 2.2. Study Design

This was a retrospective single-centre study evaluating the accuracy of Ce-MRCP in detecting and describing postoperative bile leak by comparison with PTC/ERCP, considered as a standard of reference. Factors influencing Ce-MRCP performance were also evaluated.

All patients had undergone HPB surgery and had clinical diagnosis/suspicion of BL (confirmed by abdominal drainage bilirubin levels).

All patients underwent liver Ce-MRCP to confirm the clinical suspicion of leakage and to plan further treatment, and then were studied with PTC/ERCP to confirm BL and, when possible, to deliver interventional radiological/endoscopic treatment. In all cases where trans-oral access to the biliary tract was compromised, PTC was used. Where both techniques were available, a choice was made by a multidisciplinary team including a surgeon, gastroenterologist and interventional radiologist based on the predicted BL localisation, ease of therapeutic intervention with either technique, concomitant dilatation of intrahepatic peripheral bile ducts, patient’s general conditions and specific vulnerabilities (e.g. previous acute pancreatitis, cholangitis, etc.).

The results of the Ce-MRCP investigation were compared with those of PTC/ERCP in terms of leak detection and leak localisation.

Correlations between the serum total bilirubin (TB) levels and drainage output and Ce-MRCP results were investigated.

### 2.3. Imaging Protocol

MRI examinations were performed on a 1.5 T MR system (Achieva, Philips Medical Systems^TM^, Best, The Netherlands) using a 16-channel phased-array surface coil and respiratory gating, with the patient in a supine position and fasting for at least 4 h before acquisition. Abdominal drainage was clamped closed at least 1 h before Gd-MRI. Basal imaging was acquired with T1 GRE (gradient echo) “in phase” and “out of phase” in the axial plane and half-Fourier acquisition single-shot turbo spin echo (HASTE) sequences for MRCP and multiplanar/maximum intensity projection (MPR/MIP) reconstructions specially designed to directly view the content of the biliary and pancreatic ducts.

Dynamic phases were acquired after the intravenous administration of 0.1 mL/kg of gadoxetic acid (Gadoxetate disodium, Primovist^®^; Bayer, Leverkusen, Germany), followed by a bolus of 20 mL of saline injected at a flow rate of 2 mL/s. Arterial, late arterial, portal and equilibrium phases were acquired, respectively, 15, 35, 70 and 150 s after the administration of Gd-EOB-DTPA. Subsequently, other sequences were acquired in the axial plane: T2 TSE (turbo spin-echo), T2 adiabatic spectral inversion recovery (SPAIR) and diffusion-weighted imaging (DWI). After at least 20 min, the hepatobiliary phases, recognised by the excretion of the contrast agent into the bile duct, were acquired with the use of the T1 and T1 SPGR fat-sat spoiled gradient echo (GRE) “in phase” on the axial plane. Additional delayed MR imaging at 60, 120 and 180 min was performed only if the visualisation of the biliary ducts was not sufficient for anatomical diagnosis within 30 min after the administration of Gd-EOB-DTPA. MRI images were evaluated in consensus by two experienced radiologists; specifically, for each patient, the type of surgical intervention and the possible bile duct lesion were defined, and the grade of liver parenchymal enhancement was evaluated, together with the level and timing of contrast enhancement of the biliary system.

### 2.4. PTC

PTC was performed in the angiographic suite by an experienced interventional radiologist (>10 years). The patient was placed in a supine position, the field was sterilised and local anaesthetic was injected at the puncture site before starting the procedure. Biliary system access was achieved with a combined approach, advancing a 21 G needle under US and fluoroscopic guidance. After the puncture of a subsegmentary/segmentary bile duct, a 0.018″ guidewire and 6 Fr introducer were inserted. All patients required the placement of an internal–external biliary drainage as well as further percutaneous procedures to treat the leak.

### 2.5. ERCP

ERCP was performed in a dedicated operating room by an experienced gastroenterologist (>20 years of experience). The patient was placed in a supine position and a dedicated endoscope was advanced into the second part of the duodenum. Sphincterotomy at the level of the major papilla was performed, and a retrograde cholangiography was acquired after cannulation of the common bile duct (CBD) evaluating the site of biliary leakage. Contemporary therapeutic procedures were also performed.

### 2.6. Outcome Measures

The primary outcome was the accuracy of Ce-MRCP in detecting and localising BL, defined as concordance with the results of PTC/ERCP. Bile leak was defined as per the International Study Group on Liver Surgery [12].

The secondary outcomes were the association between serum TB levels and MRCP sensitivity for BL and drainage output and MRCP sensitivity for BL. High TB levels were defined as total bilirubin > 2 mg/dL. Moreover, the coexistence of cholangitis and time for leak resolution were also evaluated.

### 2.7. Study Variables

- Data were extrapolated from a prospectively maintained database recording continuous and discrete variables regarding baseline characteristics, surgical procedure, in-hospital blood test, postoperative course and complications with a 90-day follow-up.

### 2.8. Ethics

According to the IRB of Policlinico Tor Vergata, research studies conducted retrospectively from data obtained for clinical purposes do not need ethical approval.

This study was conducted according to the international ethical recommendations on clinical research established by the Helsinki Declaration and in accordance with the STROBE criteria (htpp://strobe-statement.org, accessed on 1 January 2022) [13]

### 2.9. Statistical Analysis

Characteristics were summarised by means of the levels for categorical variables or by means of quantiles for continuous variables. Fisher’s exact test was used to analyse the correlation between the presence or absence of biliary leak and patients’ clinical laboratory parameters. In particular, bilirubin levels and drainage output were compared to define their statistical value. In all cases, a two-tailed *p* value was determined, and the null hypothesis was rejected at *p* < 0.05. The analysis was performed using R software (R Core Team (2020). R: A language and environment for statistical computing. R Foundation for Statistical Computing, Vienna, Austria. URL https://www.R-project.org/, accessed on 1 January 2022).

## 3. Results

**Figure 1:**
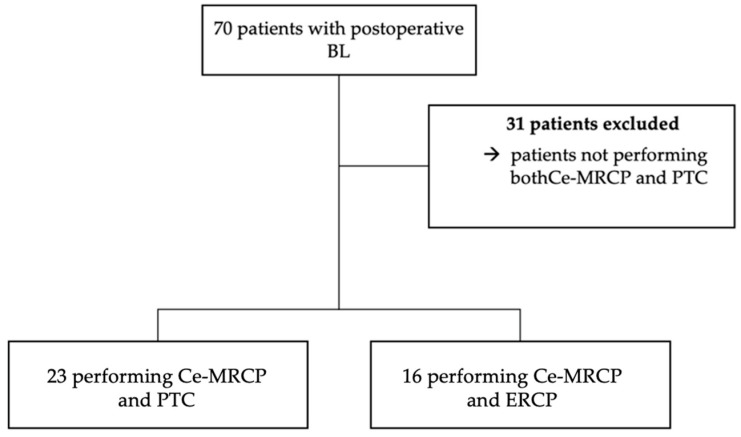
Patient selection.

Among a total of 70 patients with postoperative BL diagnosis, thirty-nine met the inclusion criteria: twenty-three undergoing Ce-MRCP and PTC and sixteen Ce-MRCP and ERCP (Figure 1).

Twenty-six patients (67%) were male, while thirteen (33%) were female. Mean age at the time of the investigation was 61 years old. Index surgical procedures included liver transplantation (38%), liver resection (26%), PD (13%) and cholecystectomy (23%). Mean time of BL onset from index surgery was 6.3 days. The BL site was the common bile duct in 62% of patients, left hepatic duct in 13%, cystic duct in 8%, right posterior hepatic duct in 5% and aberrant right posterior hepatic duct, S-IV duct, S-VII duct, right anterior hepatic duct or right hepatic duct in 3% of cases. Bile leak patients’ characteristics are summarised in Table 1.

In our study cohort, 69% of patients (27/39) were found to be positive for BL at the Ce-MRCP with excretion of Primovist^®^ in the biliary tract from 20 to 30 min after i.v. administration.

In all patients, segmentary and subsegmentary intrahepatic and extrahepatic bile ducts, as well as bilomas, were opacified using contrast medium.

Moreover, Ce-MRCP was able to detect the site of BL in all positive patients.

Lesions occurred in the CBD in 17 patients, in the left hepatic duct in 2 patients, in the right posterior hepatic duct in 2 patients, in the right anterior hepatic duct in 1 patient, in segment VII of the hepatic duct in 1 patient, in the cystic duct in 3 patients and in the aberrant right posterior hepatic duct in the remaining 1 patient. BL location results matched with those observed during PTC and ERCP in 100% of cases.

Among the twelve Ce-MRCP-negative patients, BL location was revealed at PTC in six (CBD in two patients, left hepatic duct in three patients and IV segment duct in one patient) and at ERCP in another six (CBD in five patients and right hepatic duct in one patient).

Ce-MRCP detected a bile leak in 63% and 74% of patients who were later positive at ERCP and PTC, respectively.

High levels of serum TB were found in 24 patients, and this group included the 12 patients negative on Ce-MRCP. A strong correlation between Ce-MRCP BL detection and serum TB < 4 mg/dL was found (*p*: 0.0001). Daily drainage output was not found to be correlated to BL detection with Ce-MRCP, with no particular cut-off being significant.

### 3.1. Treatment of Leak and Time for Leak Resolution

In all PTC group patients, percutaneous internal–external drainage alone or in association with other procedures (radiological–endoscopic rendezvous, plastic or covered stent placement, glue embolisation) successfully treated the leak, resulting in complete cessation of biliary output from the abdominal drainage.

In more detail, in 18 patients, internal–external biliary drainage alone was sufficient to treat the leak; biliary drainage was kept open and connected to a proper drainage bag until the biloma output drainage ended. At this time, the biliary drainage was closed. Finally, if biloma drainage remained silent and control cholangiography did not demonstrate residual leakage and/or stenosis, all the drainages were removed.

Moreover, further procedures were required in specific patients; among the 18 aforementioned patients, three required further insertion of a plastic stent to prevent stenosis at the leak level, two patients required a radiological–endoscopic rendezvous for total transection/disconnection at the level of the CBD-Luschka duct along with final positioning of two to four plastic stents, two patients with leak in the right hepatic duct and CBD were managed with the positioning of metal-covered stents and, finally, in just one patient with a peripheral low-flow leakage due to hepatic wedge resection, direct embolisation with a mixture of Glubran–Lipiodol was performed, with immediate leak resolution.

The average time for leak resolution was 14 days (range 1–45 days). One patient died during the observational period due to hepatic failure.

In all ERCP patients, a stent (plastic or metal-covered, or partially covered) was placed, resulting in complete cessation of bile leak from the percutaneous biloma drainage. In detail, 15 patients required the positioning of one or more plastic stents, while a covered metal stent was implanted in only one patient.

The average time for leak resolution was 24 days.

### 3.2. Management of Cholangitis

Human bile is normally sterile, but may become infected due to incomplete biliary obstruction or as a iatrogenic complication of operative or non-operative interventions (endoscopic or radiological). Clinical presentation can be subtle, particularly in the early phase, where abnormal liver function is the most common alteration. All patients included in this study were evaluated for the possible presence of cholangitis; complete blood count, liver function and inflammatory markers such as erythrocyte sedimentation rate (ESR), C-reactive protein (CRP) and procalcitonin (PCT) were included, as well as microbiological evaluation of the bile after drainage. Overall, among the 39 patients included in the study, 12 manifested evidence of infection. The test results are summarised in Table 2 and patients were distributed as follows: 5 showed elevated white cell count, and for inflammatory markers, CRP was elevated in 10 patients, and PCT in 9. Liver function showed alteration in 10 cases, with GGT in 8 and alkaline phosphatase in 7. Lastly, bile culture was positive in 8 patients with *E. Faecalis*, *E. Faecium*, *C. Freundii* and *C. Albicans* being the most commonly isolated.

Treatment of cholangitis was achieved using antibiotic therapy and biliary drainage (in the PTC group). A combination of ampicillin and aminoglycoside provides good cover for Gram-positive cocci and Gram-negative bacteria, respectively. In patients who had previous biliary intervention, the addiction of metronidazole to cover anaerobes can be useful. Sometimes, mezlocillin and piperacillin can also be administered due to their high bile concentration excretion. However, an effective therapy should always be based on blood or bile culture, especially in patients who have undergone previous biliary intervention. The mean time for the normalisation of blood parameters and resolution of the cholangitis (with negative bile culture) was 14 days.

## 4. Discussion

Early treatment and resolution of postsurgical biliary complications strongly depends on a rapid and accurate diagnosis. First-line investigations in most patients with suspected BL are usually US or CT. However, both modalities lack the ability to differentiate bilomas from other types of fluid collections and to eventually locate the site of BL [14].

Therefore, when BL is suspected using these modalities, usually, ERCP has to be performed to precisely identify the site of the leak and initiate treatment. ERCP has a sensitivity of more than 90% in detecting bile leaks and can also be used to demonstrate additional biliary pathology, yet it is intrinsically unable to evaluate extra-biliary structures, resulting in a significant rate of false negatives due to the non-visualisation of ducts that are disconnected from the main biliary tree [15]. Moreover, after hepatic jejunostomy in pancreatic or hepatobiliary surgery, ERCP is hardly feasible. PTC can be used to confirm and exactly locate a bile leak, but, like ERCP, is an invasive procedure that carries potential infectious complications such as cholangitis, cholecystitis, abscess formation, peritonitis and sepsis, as well as non-infectious ones such as BL and severe haemorrhage [16]. Its use should probably be limited to cases in which other modalities have failed to diagnose BL or when a simultaneous management of BL itself is indicated.

An important diagnostic contribution can, therefore, be provided by Ce-MRCP, which represents an accurate and dynamic study, providing functional as well as morphological information about biliary lesions. When Primovist is injected intravenously, it is incorporated into hepatocytes by an anionic transport system after the vascular phase. Glutathione-S-transferase has been identified as an intracellular transport protein for Gd-EOB-DTPA [17]. Excretion into the biliary tract is also mediated by the glutathione-S-transferase transport system. Intense signal enhancement of the common bile duct after intravenous administration of Primovist begins after approximately 5–16 min [2]. This method detects bile ducts by increasing the signal intensity of bile on T1-weighted images acquired during the hepatobiliary excretion of the contrast medium. The high contrast obtained in the biliary tract after contrast administration makes MPR (multiplanar reconstruction) and MIP (maximum intensity projection) of the biliary tree highly diagnostic. It has been demonstrated that bilirubin and Gd-EOB-DTPA have a high affinity for the same hepatocellular receptor [18,19].

In our study, Ce-MRCP was able to detect bile leak in approximately 66% of patients, while the remaining cases required PTC to confirm and treat the BL. In fact, as reported in other studies, adequate bile duct filling by the contrast material requires conserved liver function [20,21]. Previous studies analysed the role of Gd-MRI in the non-invasive diagnosis of biliary leak. Sensitivities reported range between 81.2 and 100%; in our experience, we reported a sensitivity of 67%. This lower value could depend either on the small sample size of the present study or on the different clinical condition of this specific cohort [2,4,7,8,9,11,22]. In fact, as reported by Tschirch et al., who conducted a study on patients with liver cirrhosis, the sensitivity of Ce-MRCP declines steadily with increasing serum TB concentrations [2]. In particular, serum TB > 1.75 mg/dL was associated with “insufficient visualisation of the biliary tree for anatomical diagnosis” in cirrhotic patients. In the present paper, all false-negative investigations were observed in patients with serum TB > 4 mg/dL, which was identified as the cut-off above which uptake of Gd-EOB-DTPA by the liver seems to be effectively absent without opacification of the biliary tree at all.

These results demonstrate that in patients with normal liver function, ce-MRCP is highly able to identify the presence and the location of the leak, approaching a sensitivity of a 100%.

As mentioned above, this could be explained by the high affinity of bilirubin for the glutathione-S-transferase transport system, which is also the intracellular transport for the Gd-EOB-DTPA in the hepatocyte. High TB levels act as a competitor binder, preventing the contrast molecule from entering hepatocytes. Alternatively, high levels of TB could simply indicate a condition of impaired liver function, resulting in the deficient excretion of Primovist in bile. The identified cut-off is higher than that found, for example, by Tschirch, but this difference may have been profoundly influenced by the patient cohort, which, in the present study, was mostly composed of non-cirrhotic patients.

**Figure 2:**
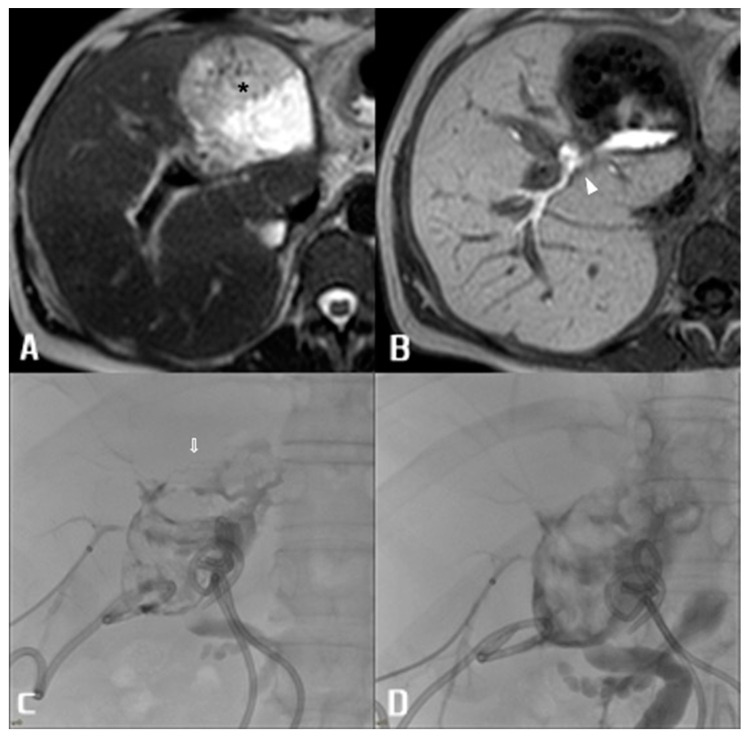
39 y.o. female patient with split right liver transplant. (**A**) T2-weighted axial image depicts large peri-hilar biloma with inhomogeneous signal (arrow). (**B**) T1-weighted images obtained after 20 min administration of c.m. show extravasation of c.m. in the biloma at the origin of the right hepatic duct (arrowhead). (**C**,**D**) Percutaneous cholangiography performed after puncture of subsegmentary duct of V segment confirmed leakage and biloma opacification with delayed advancement of c.m in the common bile duct of the recipient.

In this study, in all patients with a positive Ce-MRCP, BL location was anatomically accurate, matching the PTC/ERCP results. This may significantly influence clinical practice, as Ce-MRCP could play a fundamental role in rapidly achieving a precise anatomical diagnosis, simplifying the choice of the most appropriate treatment approach based on BL location. In turn, this could expedite the up-front delivery of the most suitable treatment for each patient’s specific condition, which may help in decreasing condition- and treatment-related complications such as cholangitis, peritonitis, sepsis and bleeding due to vascular erosion at the biloma site. Re-operative management of BL, especially in delayed diagnosis, is usually not first-line in order to avoid further surgical stress in a patient in a precarious clinical condition and with a high risk of a hostile abdomen. Therefore, PTC and ERPC have a fundamental role, and exploiting Ce-MRCP may be extremely useful. PTC can, thus, be planned with greater detail, permitting faster and better-targeted management of multi-ductal percutaneous access. Furthermore, knowledge of the precise anatomical location of BL can aid the logistics of intervention: for example, radiological–endoscopic rendezvous can be the treatment of choice for complete transection of the common biliary duct [23,24,25,26], and characterising this lesion beforehand may predict the necessity of the presence of an interventional radiologist and endoscopist (Figure 2).

**Figure 3:**
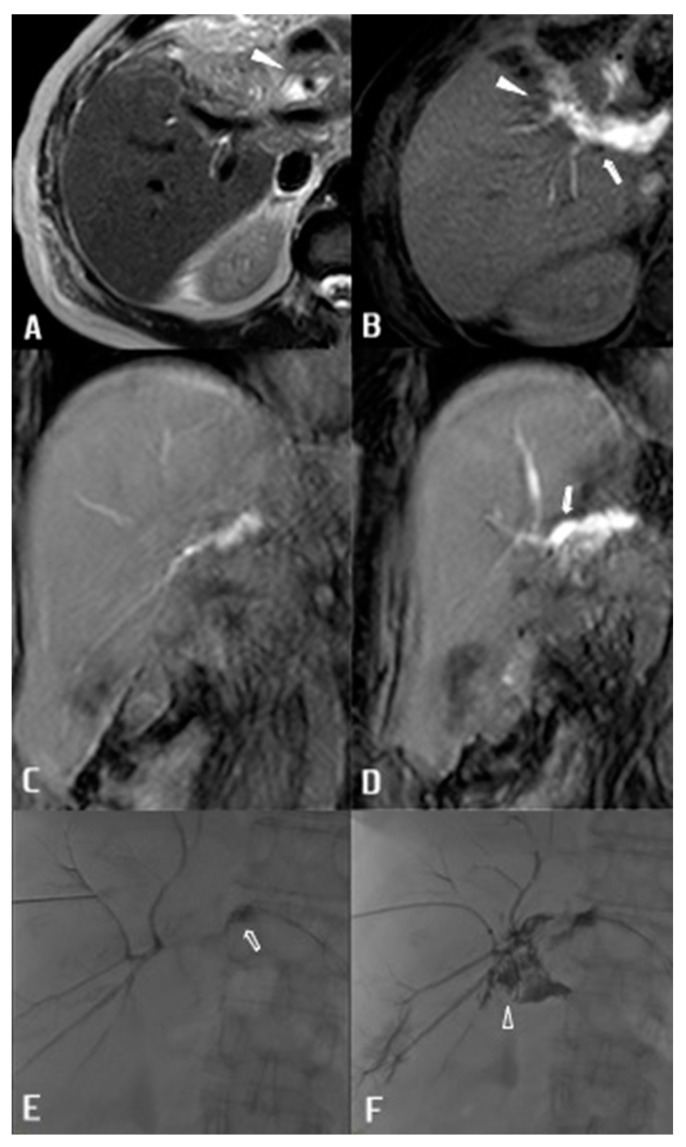
72 y.o. female patient with left hepatectomy and double bilio-enteric anastomoses. (**A**) T2-weighted axial image shows site two sites of the anastomoses with fluid collection medially to the intestinal loop (arrowhead). (**B**) T1-weighted axial image obtained 20 min after administration of c.m. demonstrates opacification of the right posterior (arrow) and right anterior (arrowhead) anastomotic ducts and opacification of the fluid collection across the surgical drainage tube. (**C**) T1-weighted coronal image acquired 20 min after administration of c.m. demonstrates right anterior anastomosis with opacification of bowel loop. (**D**) T1-weighted coronal image acquired 20 min after administration of c.m. demonstrates right posterior anastomosis with clear evidence of cm leakage (arrow). (**E**,**F**) Percutaneous cholangiography after puncture of a sub-segmentary VIII segment duct confirms leakage at the anastomotic site with precocious opacification of fluid collection across the drainage tube and delayed opacification of the intestinal loop (arrowhead).

In three cases in this series, BL developed after left hepatectomy with double bilio-enteric anastomosis for a 3b Bismuth-Corlette Klatskin tumour. Anastomoses had been performed between the jejunum and right anterior and right posterior bile ducts. Ce-MRCP was able to depict the precise location of the single-anastomosis BL, demonstrating dehiscence exclusively in the right anterior or posterior bile duct (Figure 3).

These results allowed a specific percutaneous approach leading to puncturing of the exact biliary system requiring drainage. This granted minimisation of biliary system punctures, potentially reducing complications such as arterial intrahepatic bleeding.

Another situation in which Ce-MRCP would undoubtedly be useful is in the assessment of suspected BL after cholecystectomy. In this case, identification of a Luschka duct BL could avoid further invasive treatment, as this situation usually recovers spontaneously, while identification of different injuries to the biliary tree may optimise pre-operative planning, obviating the need for invasive diagnostic modalities altogether.

In general, in view of the considerations above, the authors believe Ce-MRCP to deserve consideration in all patients with suspicion of BL without obvious cause and localisation and with TB < 4 for non-cirrhotic patients and TB < 1.5 for cirrhotic patients, to permit the non-invasive precise anatomic definition of biliary injury and consequent tailor-made treatment planning.

The most common complication reported in the study was the intercurrent onset of cholangitis.

The management of this complication was achieved mainly with antibiotics therapy. However, we believe that keeping the biliary drainage opened even after BL resolution could result in a faster normalisation of laboratory parameters and, hence, bile culture negativisation. The mean time for leak resolution was approximately 28 days for the PTC group and 24 days for the ERCP group, independently of treatment type. To the best of our knowledge, these data seem to be in accordance with the literature [5,27,28].

The current study has some limitations, including the retrospective nature and small sample size, hampering reliability of the results, and the monocentric experience, limiting their generalisability.

## 5. Conclusions

Ce-MRCP can accurately diagnose the presence of BL. Diagnostic accuracy is substantially reduced by high serum TB concentrations. These patients may benefit from up-front third-level investigations. The localisation of BL with Ce-MRCP is very precise and perfectly concordant with PTC evaluation, and this may be particularly helpful for pre-interventional planning. PTC/ERCP preserve a higher diagnostic value, especially in cases of high serum TB, yet due to their invasive nature, their first-line use is probably best reserved for patients with impaired liver function or for BLs that require expedited interventional treatment.

## Figures and Tables

**Table 1:** Bile leak patient characteristics.

Patient	Age	Gender	Index Surgical Procedure	BL Onset from Index Surgery (Days)	Serum Bilirubin Levels at the Time of BL Onset (mg/dL)	Drainage Output at the Time of Procedure (mL/Day)	BL at Ce-MRCP	BL at PCT/ERCP	BL Site
**1**	55	M	LT	11	1.1	500	Yes	Yes	CBD
**2**	84	M	LC	1	3.2	200	Yes	Yes	RHD aberrant
**3**	40	F	Right hepatectomy	2	0.6	100	Yes	Yes	LHD
**4**	70	M	LC	1	1.5	600	Yes	Yes	CD
**5**	39	F	LT	17	0.7	500	Yes	Yes	CBD
**6**	70	M	Partial liver resection	3	0.6	100	Yes	Yes	S-VII HD
**7**	72	M	Left hepatectomy	4	0.4	800	Yes	Yes	RPHD
**8**	64	F	Left hepatectomy	2	2.5	250	Yes	Yes	RPHD
**9**	66	F	LT	8	4.1	600	Yes	Yes	CBD
**10**	61	M	Partial liver resection	3	8.9	400	No	Yes	S-IV HD
**11**	68	M	Right hepatectomy	2	10	250	No	Yes	LHD
**12**	76	M	LC	5	6.7	500	No	Yes	CBD
**13**	52	F	PD	2	1.1	540	Yes	Yes	CBD
**14**	55	M	PD	7	0.9	650	Yes	Yes	CBD
**15**	56	F	Right hepatectomy	1	0.6	260	Yes	Yes	RAHD
**16**	76	M	Right hepatectomy	2	14	800	No	Yes	LHD
**17**	59	M	LT	12	8.5	650	No	Yes	CBD
**18**	72	F	PD	4	0.7	350	Yes	Yes	CBD
**19**	59	M	PD	2	0.6	600	Yes	Yes	CBD
**20**	75	F	LC	1	2.1	800	Yes	Yes	CBD
**22**	79	M	LC	2	1.1	450	Yes	Yes	CD
**22**	65	F	PD	2	1.6	200	Yes	Yes	CBD
**23**	67	F	Left hepatectomy	3	6.5	540	No	Yes	LHD
**24**	66	M	LT	12	0.5	430	Yes	Yes	CBD
**25**	56	M	LT	6	1.3	250	Yes	Yes	CBD
**26**	49	M	LT	11	38.5	650	No	Yes	CBD
**27**	55	F	LT	13	4	350	Yes	Yes	CBD
**28**	49	M	LT	16	7.2	340	Yes	Yes	CBD
**29**	59	M	LT	12	40.9	250	No	Yes	CBD
**30**	62	F	LT	11	6	250	No	Yes	CBD
**31**	58	M	LT	15	2	340	Yes	Yes	CBD
**32**	25	M	LT	18	11.4	560	No	Yes	CBD
**33**	61	M	LT	12	0.9	800	Yes	Yes	CBD
**34**	55	M	LC	2	1.9	1000	Yes	Yes	CBD
**35**	43	M	LT	13	10.3	800	No	Yes	CBD
**36**	82	M	Left hepatectomy	2	0.7	550	Yes	Yes	LHD
**37**	34	F	LC	1	1.9	280	Yes	Yes	CBD
**38**	53	M	LC	2	0.7	150	Yes	Yes	CD
**39**	75	M	LC	2	11.5	560	No	Yes	RHD
**Total**	60.6 ± 13	M 67% F 33%	LT 15/39 (38%) Liver resection 10/39 (26%) LC 9/39 (23%) PD 5/39 (13%)	6.3 ± 5.4	5.5 ± 9	446 ± 224	Yes 69%	Yes 100%	CBD 24/39 61.5% LHD 5/39 12.8% CD 3/39 7.7% RPHD 2/39 5.1% Others 5/39 12.8%

BL: bile leak; CBD: common bile duct; CD: cystic duct; HD: hepatic duct; LHD: left hepatic duct; LT: liver transplant; LC: laparoscopic cholecystectomy; PD: pancreatic duodenectomy; RAHD: right anterior hepatic duct; RHD: right hepatic duct; RPHD: right posterior hepatic duct.

**Table 2:** Ce-MRCP bile leak detection in ERCP vs. PTC patients.

Ce-MRCP 10/16 62.5%	ERCP 16/16 100%
Ce-MRCP 17/23 74%	PTC 23/23 100%

## Data Availability

Data supporting reported results can be found in the database of Policlinico Tor Vergata (www.ptvonline.it, accessed on 1 January 2023). Data are protected and access availability must be obtained.
